# The Imported Relapsing Fever Case Was Not the First Case in Japan

**DOI:** 10.4269/ajtmh.24-0239

**Published:** 2024-06-25

**Authors:** Morimasa Yagisawa, Patrick J. Foster

**Affiliations:** Kitasato University Omura Satoshi Memorial Institute, Tokyo, Japan; Louis Pasteur Center for Medical Research, Kyoto, Japan E-mail: yagisawa2013@gmail.com; Keio University Faculty of Pharmacy, Tokyo, Japan E-mail: pjf@keio.jp

Dear Editor,

We are writing regarding an article entitled “*Case Report: The First Case of Imported Relapsing Fever in Japan*” published in this journal in 2013.^1^ Although it has been more than 10 years since the article was published, we propose a correction to clarify an inaccuracy in the article.

The case report published does not appear to be the first case of relapsing fever in Japan. To the best of our knowledge, the first report of relapsing fever in Japan was published in an official Gazette, September 21, 1895 (Japanese calendar year Meiji 28), stating, "*A Sudden Outbreak of Relapsing Fever in the Tokyo Metropolitan Geriatric Hospital caused a Temporary Outbreak of 20 Patients*.”^2^ That being said, in the lecture record of Prof. Shibasaburo Kitasato, it is explained that this Tokyo outbreak was actually the second case, with the first noted in the spring of that same year at Hiroshima Army Hospital, in a soldier returning from the Sino-Japanese War.^3^

Dr. Taichi Kitajima, the second director of the Kitasato Research Institute, investigated relapsing fever and made a field inspection in Kobe City, diagnosing 364 patients and confirming an estimated 6,000–7,000 cases in Kagawa Prefecture, as reported in the *Japanese Journal of Bacteriology* in 1896. In this report, Dr. Kitajima stated that most of the cases reported as typhoid fever were actually diagnosed as relapsing fever, and it was assumed that the source of the disease was the Sino-Japanese War that broke out two years prior. These patients, who served in the army or worked as laborers, contracted the disease in mainland China and then spread it through lice after returning to Japan.^4^

Later, during the Russo-Japanese War (1904–1905) and World War I (1914–1918), there are records of people returning to Japan from mainland China suffering from relapsing fever.^5^ From the Japan–China Incident that began in 1937 to World War II (1939–1945), a large number of people returning from mainland China, including Manchuria, suffered from relapsing fever.

The fact that postwar repatriates from mainland China and other areas controlled by the Soviet Union suffered from relapsing fever is also supported by the historical record documented by the Imperial Ordinance of 1946, forbidding the embarkation of patients with relapsing fever on repatriation vessels.^6^

In Japan, the former "Communicable Diseases Prevention Law” (enacted in 1897), stipulated 11 legal contagious diseases (including smallpox, plague, diphtheria, cholera, typhoid fever, and dysentery) and 12 reportable contagious diseases (including anthrax, measles, tetanus, rabies, malaria, yellow fever, and relapsing fever). Under the provision of the law, a monthly report, “Statistics of Communicable Disease” was published. Such monthly reports began as early as 1880 and were reorganized in 1947, but statistics for relapsing fever cases were initiated only in 1956. Because of this, relapsing fever cases diagnosed among returnees from mainland China prior to 1955 were not listed in the published statistics.^7^

Relapsing fever (classic-type) is caused by *Borrelia* species carried by lice. Such lice were eradicated in Japan by the mid-1950s with the widespread use of insecticides DDT (dichlorodiphenyltrichloroethane) and BHC (benzene hexachloride), under the orders of the occupying forces that ruled Japan until 1952. Relapsing fever also became easily treatable with injectable penicillin G (benzylpenicillin, marketed in November of 1948), orally active aureomycin (chlortetracycline, in January of 1951), and terramycin (oxytetracycline, in October of 1952). It is therefore not surprising that relapsing fever has not been documented in Japan since 1956. However, it is important to note that, in earlier years, imported relapsing fever was frequently diagnosed in Japan.

## References

[1] Kutsuna S Kawabata H Kasahara K Takano A Mikasa K, 2013. Case Report: The first case of imported relapsing fever in Japan. Am J Trop Med Hyg 89: 460–461. 23857020 10.4269/ajtmh.13-0187 PMC3771281

[2] Patients of relapsing fever , 1895. *Official Gazette No. 3671* (in Japanese). Available at https://dl.ndl.go.jp/pid/2946946. Accessed March 25, 2024.

[3] Nakagawa A, 1896. Lectures on contagious disease research by Professor Shibasaburo Kitasato. *Institute of Contagious Diseases* (in Japanese). Available at https://dl.ndl.go.jp/pid/835313. Accessed April 3, 2024.

[4] Kitajima T, 1896. Relapsing fever in Kobe City, Hyogo Prefecture and Kagawa Prefecture (in Japanese). Jpn J Bacteriol 7: 467–475.

[5] Saito Y, 1935. Clinical observations on Manchurian relapsing fever (in Japanese). J Jpn Assoc Contagious Dis 9: 613–624.

[6] Concerning Imperial Ordinance No. 311 of 1946 , 1947. *Official Gazette No. 6009* (in Japanese). Available at https://dl.ndl.go.jp/pid/2962523. Accessed March 25, 2024.

[7] Japan Ministry of Health and Welfare , 1981. *Statistics of Communicable Disease* (in Japanese). Available at https://www.niid.go.jp/niid/images/epi/archives/surveillance_nepou/densen/009_S56-S59.pdf. Accessed March 25, 2024.

